# Corticolimbic circuitry as a druggable target in schizophrenia spectrum disorders: a narrative review

**DOI:** 10.1038/s41398-024-03221-2

**Published:** 2025-01-24

**Authors:** Abigail Gee, Paola Dazzan, Anthony A. Grace, Gemma Modinos

**Affiliations:** 1 Department of Psychological Medicine, Institute of Psychiatry, Psychology and Neuroscience, King’s College London, London, United Kingdom; 2 Departments of Neuroscience, Psychiatry and Psychology, University of Pittsburgh, Pittsburgh, PA USA; 3 MRC Centre for Neurodevelopmental Disorders, King’s College London, London, UK

**Keywords:** Schizophrenia, Clinical pharmacology

## Abstract

Schizophrenia spectrum disorders (SSD) involve disturbances in the integration of perception, emotion and cognition. The corticolimbic system is an interacting set of cortical and subcortical brain regions critically involved in this process. Understanding how neural circuitry and molecular mechanisms within this corticolimbic system may contribute to the development of not only positive symptoms but also negative and cognitive deficits in SSD has been a recent focus of intense research, as the latter are not adequately treated by current antipsychotic medications and are more strongly associated with poorer functioning and long-term outcomes. This review synthesises recent developments examining corticolimbic dysfunction in the pathophysiology of SSD, with a focus on neuroimaging advances and related novel methodologies that enable the integration of data across different scales. We then integrate how these findings may inform the identification of novel therapeutic and preventive targets for SSD symptomatology. A range of pharmacological interventions have shown initial promise in correcting corticolimbic dysfunction and improving negative, cognitive and treatment-resistant symptoms. We discuss current challenges and opportunities for improving the still limited translation of these research findings into clinical practice. We argue how our knowledge of the role of corticolimbic dysfunction can be improved by combining multiple research modalities to examine hypotheses across different spatial and temporal scales, combining neuroimaging with experimental interventions and utilising large-scale consortia to advance biomarker identification. Translation of these findings into clinical practice will be aided by consideration of optimal intervention timings, biomarker-led patient stratification, and the development of more selective medications.

## Introduction

**Fig. 1:**
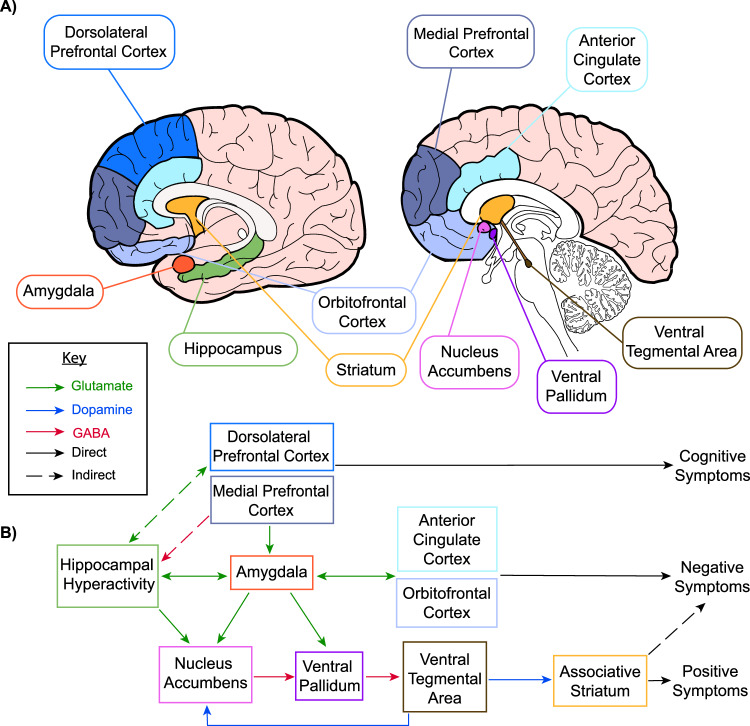
Model of how corticolimbic brain circuit dysfunction may contribute to positive, negative and cognitive symptoms in schizophrenia spectrum disorders (SSD) as adapted from previous models by Grace and colleagues. **A** Abnormalities have been identified in corticolimbic structure, functional activation and connectivity in SSD. **B** Diagram displaying a model for interactions between corticolimbic brain circuits and mechanisms leading to symptom domains. Hippocampal hyperactivity and alterations of rhythmicity cause downstream effects within the nucleus accumbens, ventral pallidum and ventral tegmental area, resulting in increased striatal dopamine signalling and positive symptoms. It may also cause negative symptoms by affecting emotional processing and reward outcomes via the amygdala, and impacting decision-making through the medial prefrontal, anterior cingulate and orbitofrontal cortices. Increased excitatory output from the hippocampus to the dorsolateral prefrontal cortex may lead to reduced synaptic density and disruption to the oscillatory activity necessary for cognitive functions.

Schizophrenia spectrum disorders (SSD) encompass a broad range of symptoms beyond the commonly recognised positive symptoms (i.e. hallucinations and delusions), affecting cognitive and negative domains. Understanding the neural circuitry involved in the development of these symptoms is crucial for improving treatments and long-term outcomes. The corticolimbic system is an interacting set of cortical and subcortical brain regions, including the medial prefrontal cortex (mPFC), anterior cingulate cortex (ACC), nucleus accumbens, hippocampus and amygdala [1] (Fig. 1). This circuitry is critical for facilitating interactions between emotion, cognition and decision-making functions, all of which are affected in SSD [2].

Recent advancements in neuroimaging have significantly enhanced our understanding of the corticolimbic system’s role in SSD. For instance, ultra-high field magnetic resonance imaging (MRI) offers precise examination of corticolimbic structure, function and connectivity at subfield levels [3]. Moreover, higher-resolution imaging enhances resolution for magnetic resonance spectroscopy (MRS), facilitating more reliable and specific measurements of relevant metabolites [4]. Multimodal imaging can combine structural, functional and molecular data, yielding a more comprehensive view of corticolimbic dysfunction across different spatial and temporal scales [5]. Additionally, large-scale consortia that share multimodal data address the limitations encountered in previous studies, such as small sample sizes, heterogeneous protocols and reproducibility issues [6, 7]. These large-scale studies provide the required power for multivariate data-driven methods, such as machine learning, allowing inferences to be made at the subgroup [8] or individual level [9], helping to circumvent the limitations of traditional analytic approaches that compare patients to healthy controls at the group level to untangle clinical and biological heterogeneity in SSD. Another innovative approach is normative modelling, which involves comparing individuals to a reference model in order to make inferences at the individual level [10]. Integrating these methodologies enriches our understanding of corticolimbic dysfunction and holds promise for a more personalised approach to SSD management by identifying biomarkers at the individual level. Furthermore, combining advanced neuroimaging methods with drug administration studies has the potential to translate corticolimbic system findings into clinically relevant therapeutic targets. This approach uses pharmacological agents to target specific mechanisms and analyses the impact on broader activation and signalling pathways [11]. This enhances our comprehension of the neurobiological mechanisms underlying SSD symptoms and has promise in predicting individual medication response [11].

To achieve a better understanding of the mechanisms leading to positive, negative and cognitive symptom manifestations, this review synthesises recent developments in the study of corticolimbic dysfunction in the pathophysiology of SSD with a focus on neuroimaging advances and approaches to bridge evidence across different scales of description (cellular/molecular, circuit, whole-brain imaging and behaviour). Finally, the discussion addresses how these methodologies and findings can be leveraged to inform the identification of novel therapeutic and preventative targets in SSD.

## Mechanisms of corticolimbic dysfunction in SSD

The corticolimbic system relies on coordinated interactions between the PFC and limbic regions. The hippocampus and amygdala are highly interconnected limbic structures located in the medial temporal lobe which act at the interface between cognition and emotion [12]. The hippocampus plays a key role in memory and associative learning [1], while the amygdala is involved in processing emotion and biological salience [1]. These structures bidirectionally influence each other, with the amygdala modulating hippocampal-dependent memory encoding and consolidation for emotional stimuli, and the anterior hippocampus regulating the amygdala’s response to emotional stimuli [12]. The PFC integrates sensory and limbic inputs to regulate cognitive-emotional processes and guide goal-directed behaviours [2]. Disruptions to multiple regions in these complex circuits contribute to the emotional and cognitive dysregulation observed in SSD.

Among these regions, the hippocampus has been consistently identified as a central hub of abnormality within the corticolimbic system in SSD [13, 14]. Neuroimaging studies have demonstrated reductions in volume [15], and increases in resting cerebral blood volume [16] and flow [17, 18]. Resting hippocampal hyperactivity has also been found in individuals at clinically high risk for psychosis (CHR-P) [19–21] and healthy people with psychosis-like experiences [22]. This resting hyperactivity impacts function as there is reduced task-related recruitment of the anterior hippocampus during memory and visual stimulation in early psychosis [17]. Hippocampal hyperactivity has also been associated with negative and cognitive symptom severity [23], and predicts transition to psychosis in CHR-P [24, 25]. In terms of connectivity, reduced resting functional connectivity has been identified between the hippocampus and limbic and frontal regions across the SSD spectrum [26], indicating broader changes to corticolimbic circuitry.

Regarding mechanisms underlying corticolimbic dysfunction, several lines of evidence suggest it results from an imbalance in excitatory and inhibitory signalling [27]. A balanced interplay between glutamatergic neurons and gamma-aminobutyric acid (GABA) interneurons, termed excitation/inhibition balance, is required for the development of microcircuits and larger neural networks [27]. There are several families of glutamatergic receptors, including N-methyl-D-aspartic acid (NMDAR), α-amino-3-hydroxy-5-methylisoxazole-4-proprionic acid (AMPAR), kainite and metabotropic receptors (mGluR). There are also diverse GABAergic interneuron cell-types, identified by the presence of calcium-binding proteins (e.g. calbindin, parvalbumin), neuropeptides (e.g. somatostatin, cholecystokinin) or morphological appearance (e.g. chandelier, basket) [28]. This complexity leads to multiple possible mechanisms that may disrupt excitation/inhibition balance and, consequently, hippocampal and broader corticolimbic system disruption.

Among GABAergic interneuron cell-types, parvalbumin-expressing interneurons (PVI) are key to maintaining excitation/inhibition balance and shaping cortical circuitry during development [29]. Alterations in PVI are well-established in SSD by post-mortem examination, including decreased density of PVI in the hippocampus [30, 31] and decreased parvalbumin protein/mRNA expression in the dorsolateral PFC (dlPFC) [32]. NMDAR hypofunction has been implicated in these alterations, with pre-clinical evidence that NMDAR disruption can lead to oxidative stress [33] and reduced PVI density in the hippocampus and PFC [34]. Parvalbumin is a calcium-binding protein which enables high frequency firing by shortening the post-spike after hyperpolarisation [35]. However, this high metabolic demand renders these neurons more susceptible to oxidative stress [36] (an imbalance between free radicals and antioxidant defence). Severe long-term imbalances can result in cell damage and death [37], while smaller imbalances reversibly alter the function of redox-sensitive proteins, impacting neurotransmission, cellular proliferation and differentiation [38]. Several interacting factors lead to feedforward loops exacerbating oxidative stress; this includes: NMDAR hypofunction [39], antioxidant deficits [40], mitochondrial dysfunction [41] and neuroinflammation [42]. These cascading effects, if not compensated, can lead to progressive hippocampal dysfunction and broader corticolimbic abnormalities [37].

## Consequences of corticolimbic dysfunction

Corticolimbic system abnormalities are proposed to drive diverse symptom domains in SSD [43, 44] (Fig. 1). More specifically, hippocampal hyperactivity causes downstream effects within the nucleus accumbens, ventral pallidum and ventral tegmental area, resulting in increased striatal dopamine signalling and positive symptoms. Additionally, hippocampal hyperactivity and dysrhythmicity may lead to negative symptoms through disruptions of amygdala activity, impacting emotional salience and reward processing, and with the mPFC, ACC and orbitofrontal cortex, impacting reward anticipation and value-based decisions. Finally, increased excitatory output from the hippocampus indirectly to the dlPFC may lead to disruption of the oscillatory activity critical for cognitive functions. This section explores the neuroimaging evidence for this model and explores how this may lead to different SSD symptom domains.

### Positive symptoms

Positive symptoms have been linked extensively to the dopamine system: elevated pre-synaptic striatal dopamine synthesis capacity and release is a robust finding in SSD, and all current antipsychotics target dopamine D2 receptors to some degree [45]. Numerous pre-clinical studies ([44] for overview) have examined the involvement of corticolimbic circuitry in dysregulating dopaminergic system function. Here, we provide a brief overview of these findings focusing on neuroimaging evidence.

Pre-clinical studies suggest that the spontaneous activity of midbrain dopamine neurons is regulated by corticolimbic regions [44], which is important for controlling the response to context and environmental conditions. Dopaminergic signalling can be tonic or phasic in nature, and these two forms of signalling are highly interlinked [44]. Tonic signalling refers to baseline spontaneous firing, which is regulated by glutamatergic afferents from the hippocampus and amygdala [46]. Phasic signalling refers to rapid bursts of action potentials which is important for determining salience and reinforcement of learning [47]. Phasic signalling relies on prior depolarisation from tonic activity [48]. For instance, in threatening contexts, the hippocampus causes a downstream increase in tonic dopaminergic activity, which causes greater phasic dopamine release and rapid focus on stimuli [47]. In SSD, disrupted modulation of the dopaminergic system may result in excessive tonic activity leading to higher phasic signalling, resulting in hyper-responsivity to irrelevant stimuli and impaired salience detection [49].

Complex interactions between multiple corticolimbic regions are involved in regulating tonic dopaminergic signalling. For instance, ventral tegmental area neurons are sustained in a hyperpolarised state by ventral pallidum GABAergic inputs, which keeps them below the firing threshold [50]. This is regulated by excitatory projections from the hippocampus to the nucleus accumbens [51], which increases inhibition of the ventral pallidum and, therefore, increases dopaminergic signalling from the ventral tegmental area to the associative striatum [52]. The amygdala activates the ventral pallidum, reducing dopamine activity between the ventral tegmental area and ventral striatum [43]. Imaging studies have investigated the functional alterations that contribute to the dysregulation of this intricate system, disrupting dopaminergic signalling and leading to aberrant salience.

Inappropriately attributing salience to neutral or irrelevant stimuli has been demonstrated across SSD stages and is a contributing mechanism in the formation of positive symptoms [53]. Imaging studies using salience and reward prediction error paradigms have identified functional alterations in the corticolimbic system in SSD. For instance, in oddball paradigms, antipsychotic-naïve first-episode psychosis patients showed reduced activation in the amygdala, VTA, ACC and striatum in response to negative emotional salience [54]. Similar findings are seen in CHR-P individuals using the same paradigm [55]. A positron emission tomography (PET) study using a [^15^O]H_2_O tracer with emotionally salient visual images reported impaired ventral striatum response to salient stimuli and elevated baseline activity in the ventral striatum and the amygdala, which inversely correlated with overall symptom severity [56]. Finally, prediction error, the mismatch between predicted and actual outcomes, is closely linked to salience. Studies with reward prediction error paradigms reveal abnormalities in the striatum, midbrain, limbic regions and mPFC in first-episode psychosis [57, 58] and CHR-P [59]. Given its association with reward processing and negative symptoms, we discuss this further in the following section.

PET studies using radiolabelled L-dihydroxyphenylalanine (L-DOPA), the dopamine precursor, have reported increased dopamine synthesis capacity in the associative striatum in CHR-P individuals [60, 61] and those with SSD [62]. A study with SSD individuals during a relapse of psychotic symptoms found increased intrinsic activity in the dorsal striatum, which correlated with positive symptom severity [63]. Multimodal imaging combining PET and MRS in CHR-P individuals found a negative correlation between hippocampal glutamate levels and striatal dopamine synthesis capacity, this was particularly marked in the CHR-P individuals who later transitioned to psychosis [64]. This suggests that the relationship between hippocampal glutamate and striatal dopamine may be a risk marker for psychosis transition in CHR-P.

Disrupted control of dopamine signalling due to excessive phasic release may result in diminished functional connectivity between the cortex and the associative striatum, thereby impairing integration from emotional, cognitive and motor areas [65]. fMRI studies have found decreased functional connectivity between the associative striatum and the ACC and dlPFC in psychosis-like experiences [66], CHR-P [67], first-episode psychosis and SSD [68]. Interestingly, improvements in positive symptoms during antipsychotic treatment have been associated with increased functional connectivity between the striatum and the ACC, dlPFC and limbic regions [69]. This improved functional connectivity may provide a biomarker for symptom improvements with antipsychotic treatment.

There is evidence that the dopamine system also contributes to cognitive and negative symptoms. Increased striatal dopamine and disrupted connectivity between the frontal cortex and associative striatum and/or disruption of cortical dopamine may contribute to cognitive impairments [70]. The striatum regulates reward-related behaviours, and impaired reward processing is associated with negative symptoms [71, 72], as discussed in the following section.

### Negative symptoms

Negative symptoms have been classified into five key constructs within two overarching factors: diminished expression (blunted affect and alogia) and amotivation (asociality, avolition and anhedonia) [73]. Approximately 40–60% of patients with SSD experience prominent negative symptoms, which are also frequently present in early psychosis and in CHR-P [74]. Multiple corticolimbic regions have been associated with negative symptoms, including the hippocampus, amygdala, mPFC, orbitofrontal cortex, ACC, striatum and insula [75, 76]. Depending on the specific location of dysfunction within these circuits, a range of symptomatology may be explained, including diminished expression and amotivation.

The hippocampus regulates affective states and shares strong reciprocal connectivity with the amygdala, a key structure in emotion perception [77], emotional significance processing [78], emotional regulation [79] and salience evaluation [80]. Neuroimaging studies consistently implicate the amygdala in SSD, showing reduced volumes in multiple meta-analyses [81, 82] and in large consortium studies [83, 84]. Lower amygdala volume is observed in CHR-P individuals and in unaffected relatives [85, 86], suggesting a potential neurodevelopmental and/or genetic origin. There are alterations in structural and resting functional connectivity between the amygdala and corticolimbic regions. This includes reduced fractional anisotropy in the uncinate fasciculus [87], linking the amygdala to the mPFC/ orbitofrontal cortex, although some studies have only found this in patients with prominent negative symptoms [88]. Age-related alterations in amygdala resting functional connectivity reveal a nuanced trajectory: during late childhood and early adolescence, there is reduced functional connectivity with the putamen/occipital cortex [89], subsequently there is an accelerated decline in functional connectivity with the ventrolateral PFC, striatum and thalamus [89]. Reductions in resting functional connectivity between the amygdala and orbitofrontal cortex have been observed across SSD stages, and this correlates with overall symptom severity [87, 90]. Meta-analyses have found that orbitofrontal cortex cortical thinning is associated with negative symptom severity [91].

Functional neuroimaging studies examining cognitive-emotional processing in SSD have primarily used facial emotion recognition tasks. There is evidence for facial emotion processing dysfunction in first-episode psychosis [92], in SSD [93] and in first-degree relatives [94]. However, findings in CHR-P have been mixed with some studies finding a significant difference for emotion identification but not discrimination [95], and others finding poorer recognition only for negative emotions [96]. Meta-analyses have demonstrated reduced amygdala activation in response to emotional stimuli in first-episode psychosis [97] and in chronic SSD [98, 99]. The findings in CHR-P have again been inconsistent [97], possibly due to sample heterogeneity and methodological inconsistencies. Reduced amygdala activation represents reduced recruitment when processing emotionally salient stimuli and/or increased response to neutral stimuli. Dysfunctional PFC engagement, notably the ACC and dlPFC, is evident in SSD and psychosis risk during tasks involving emotional stimuli appraisal [100], subjective affect regulation [101] or inhibition of emotional distractions [102].

The “emotional paradox” in SSD describes a disconnect between reported emotional experiences and observed behaviours [103]. While individuals with SSD often report intact emotional experiences [104], studies indicate reduced engagement in pleasurable activities [105]. Functional neuroimaging, particularly using paradigms such as the Monetary Incentive Delay task [106], has examined neural responses to reward anticipation and outcomes. While most studies report reduced ventral striatum activation during anticipatory reward [107], findings are inconsistent and are influenced by antipsychotic medication and negative symptom severity [108, 109]. Neural responses to reward outcomes vary, with some studies showing reduced activation in the ventral striatum, mPFC, and orbitofrontal cortex [109, 110], while others find no significant differences [111]. A recent meta-analysis found reduced activation in the ACC, amygdala, insula and striatum during anticipation [112]. Striatal activation decreases correlated with increased negative symptoms [112]. During reward outcomes, there was increased activation in the striatum, insula, amygdala, hippocampus and right parahippocampal gyrus, alongside decreased activation in the mPFC and dlPFC [112]. Decreased mPFC activation correlated with increased positive symptoms [112].

Deficits in value-based decision-making may be due to difficulty integrating information and updating value estimations [113]. Studies using probabilistic selection paradigms have revealed reinforcement learning deficits in SSD [72]. Interestingly, while preference for previously rewarded stimuli was reduced, patients could learn to avoid stimuli associated with negative outcomes, and this was particularly pronounced in those with severe negative symptoms [72]. Compared to healthy controls, SSD participants have difficulty integrating value information when making behavioural choices [71]. In controls, valued actions were associated with increased caudate and mPFC activation, whereas patients exhibited significantly less caudate activity, which correlated with measures of avolition and alogia [71].

Overall, the evidence suggests that corticolimbic brain changes including the amygdala, hippocampus, PFC and striatum lead contribute to negative symptoms in SSD by impairing emotional processing and motivation.

### Cognitive deficits

Cognitive dysfunction in SSD impacts multiple cognitive domains, often precedes psychosis onset [114] and is closely associated with poorer social and occupational functioning [115]. The PFC has a well-established role in supporting higher-order cognitive processes. Studies have examined the cellular and local network changes which may result in altered regional PFC activity and impaired recruitment of wider task-relevant networks, resulting in cognitive symptoms.

The PFC receives dense glutamatergic projections from the hippocampus [116]. Hippocampal hyperactivity, therefore, increases excitatory input to the PFC and this increased tonic activity can lead to reductions in synaptic density [117]. For instance, post-mortem analyses in SSD have shown reduced density of basilar dendritic spines on layer III pyramidal neurons in the dlPFC [118] and decreased synaptophysin protein, a marker of axon terminals, in the PFC and hippocampus [119].

Gamma oscillations are a potential translational bridge between these cellular/molecular alterations and cognitive symptoms. The coordinated firing of neuronal populations creates rhythmic brain activity, which is reflected as oscillations in extracellular brain potentials [120]. In SSD, electroencephalogram (EEG) and magnetoencephalography (MEG) has shown differences in oscillatory activity during cognitive tasks, including in theta, alpha and gamma ranges [121, 122]. In particular, impaired task-evoked gamma oscillatory activity has been found during cognitive control [123, 124] and working memory tasks [125, 126] in first-episode psychosis and chronic SSD, regardless of medication status.

Synaptic interactions between interneurons and pyramidal cells are involved in establishing and maintaining brain oscillations [127]. PVI strongly impact gamma modulation in pre-clinical studies [128]. Excitation of PVI by pyramidal neurons provides coordinated feedback inhibition to multiple pyramidal neurons [120]. Following inhibition, pyramidal neurons are more likely to fire in coordination, thereby generating oscillations [120]. PVIs also synapse onto themselves, which may help synchronise oscillations [129].

Understanding the mechanisms underlying gamma oscillation alterations and their cognitive consequences is a focus of pre-clinical and human research. Optogenetics, involving the introduction of light-sensitive proteins into specific cells to regulate their activity (for review of methods, see [130]), has demonstrated that inhibiting PVI in the PFC supresses gamma oscillations [131]. Rhythmic light stimulation at the gamma frequency enhanced interneuron-driven gamma oscillations and normalised rule-shifting task performance [132]. Furthermore, misaligned light stimulation disrupts gamma synchronisation and impairs task performance [133]. In humans, transcranial direct current stimulation, which modulates cortical excitability via low-current scalp electrodes, increases gamma oscillatory power and enhances cognition when applied to the dlPFC [134]. Collectively, these findings suggest that modifying PFC interneuron function alters oscillatory activity and impacts cognitive function.

PFC GABA and glutamate function in SSD has been assessed using neuroimaging techniques. MRS meta-analyses found lower glutamate in the mPFC [135, 136] but no significant difference in GABA levels in the mPFC or dlPFC [137]. A large meta-analysis reported higher glutamate to creatine ratio in the mPFC; this was positively associated with increased total symptom severity and antipsychotic dose was negatively associated with mPFC glutamate levels [138]. This indicates that higher levels of glutamate in the mPFC are associated with higher symptom severity but that these levels may be reduced with antipsychotic medication. While higher GABA levels predicted better working memory performance in healthy controls [138], the opposite was found in SSD [139]. This suggests that GABAergic increases may serve a compensatory role for disrupted connectivity between inhibitory interneurons and pyramidal cells. A small EEG and MRS study found that peak gamma frequency during the encoding stage of a working memory task correlated with dlPFC GABA levels and task performance [125]. While MRS can provide regional quantification of biochemistry in vivo, this approach measures extracellular and intracellular metabolite concentrations across all tissue types. Hence, these findings cannot provide higher-resolution insights into neurophysiological changes.

PET can be used to measure GABA regulation with benzodiazepine site-binding radiotracers (e.g. [^11^C]flumazenil). In healthy participants, [^11^C]flumazenil binding increased after blockade of the GABA membrane transporter with tiagabine, which significantly correlated with gamma oscillatory power during a working memory task [140]. Another study reported positive correlations between tiagabine-induced change in binding and delay-related gamma oscillatory power in healthy participants, whereas SSD participants exhibited reduced capacity to increase extracellular GABA [141]. This reduced capacity was associated with decreased gamma oscillatory power during the cognitive task, particularly in antipsychotic-naïve individuals. Baseline [^11^C]flumazenil binding was raised in antipsychotic-naïve individuals, possibly reflecting early compensatory GABA_A_ receptor upregulation.

Overall, findings point to the key role of hippocampal hyperactivity in increasing excitatory input to the PFC, causing cellular, molecular and network alterations. Gamma oscillations have emerged as a translational link between these alterations and cognitive symptoms, with disruption observed across SSD stages. Neuroimaging studies investigating PFC GABA and glutamate reveal associations with gamma oscillatory power, treatment response and cognitive deficits. These findings underscore the intricate relationship between these neurophysiological alterations and cognitive dysfunction in SSD.

## Targeting corticolimbic circuit dysfunction

There is a persisting unmet need to develop new therapeutic options to treat the negative, cognitive and treatment-resistant symptoms of SSD, and the corticolimbic system shows promise as a target for upstream interventions. This section outlines investigated targets and the effects on molecular and wider circuit functioning via neuroimaging modalities.

### Targeting excitation/inhibition balance

**Fig. 2:**
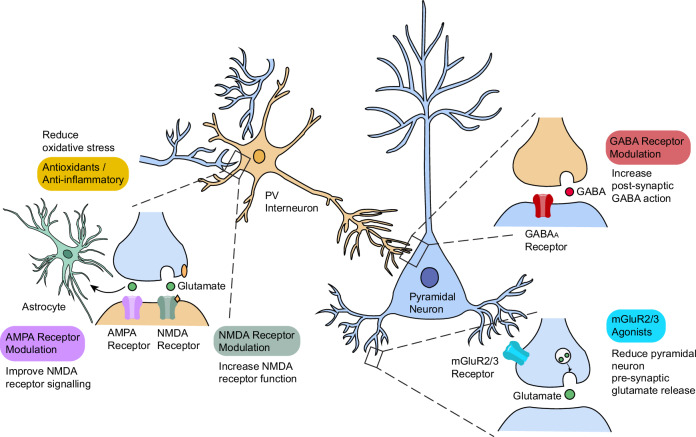
Diagram displaying interaction between pyramidal neurons and parvalbumin interneurons (PVI) and possible targets for intervention in schizophrenia spectrum disorders (SSD). PVI primarily exert inhibitory effects via synapses onto the perisomatic region of pyramidal cells. Gamma-aminobutyric acid (GABA_A_) receptors on the postsynaptic membrane receive GABA signalling from the PVI. A possible mechanism to compensate for the loss of PVI function is to increase postsynaptic GABA transmission via GABA_A_ receptor modulation. PVI receive input via N-methyl-D-aspartic acid receptors (NMDAR) and α-amino-3-hydroxy-5-methylisoxazole-4-proprionic acid receptors (AMPAR). Another possible mechanism leading to excitation/inhibition imbalances is the hypofunction of NMDAR on PVI, leading to reduced inhibition of pyramidal cells and increased glutaminergic transmission. A potential method to correct this is to increase the activity of the NMDAR. Allostatic modulation of AMPAR facilitates the removal of magnesium from NMDAR and, therefore, may also improve NMDAR signalling. Another possible approach is to reduce pre-synaptic glutamate release from pyramidal cells via positive allostatic modulation of metabotropic glutamate receptors (mGlu2/3). Finally oxidative stress can lead to dysfunction and loss of PVI, therefore antioxidants and anti-inflammatory agents have been investigated in SSD.

As discussed above, deficits in hippocampal PVI and increased pyramidal neuron activity can lead to excitation/inhibition imbalances across the network. Pharmacological approaches to rebalance excitation/inhibition include modulation of glutamatergic or GABAergic mechanisms (Fig. 2) [142].

#### GABAergic mechanisms

Enhancing postsynaptic GABA transmission is one strategy to compensate for the loss of PVI function. There are ionotropic GABA_A_ and metabotropic GABA_B_ receptors, each with diverse subunits and uneven tissue distribution [143].

Broad modulators like benzodiazepines act on multiple GABA_A_ subunits, which can lead to sedation, tolerance and dependence. An observational cohort study found that benzodiazepine exposure in CHR-P individuals was not associated with a significant change in psychosis transition risk after controlling for confounding by indication [144]. A single dose of diazepam significantly reduced hippocampal blood flow in CHR-P individuals [145], aligning with pre-clinical evidence suggesting that enhancing GABA signalling can reduce hippocampal/amygdala hyperactivity, by attenuating hippocampal parvalbumin loss [146, 147]. Clinical application in SSD requires further investigation and will benefit from the use of more selective medications.

GABA_A_-α5 subunits are abundant in limbic areas and regulate inhibitory input to pyramidal neurons without impacting sedation [148]. PET studies have demonstrated lower α5 subtype GABA_A_ receptor availability in the hippocampus of antipsychotic-free patients with SSD [149]. In animal models, selective GABA_A_-α5 positive allosteric modulators reversed ventral hippocampal hyperactivity [150]. An α2/3 selective GABA_A_ medication (MK-0777/TPA023) showed promise in enhancing frontal gamma activity and improving cognitive performance in psychosis [151], but there was no significant cognitive benefit in a larger trial [152].

Potassium channels are extensively expressed on PVI, allowing them to fire at the high frequencies needed to synchronise pyramidal cells. Kv3-type voltage-gated potassium channels are highly expressed in corticolimbic brain circuits [153]; they have been shown to improve PVI firing [154] and rescue hippocampal network synchrony in vitro [155]. In animal models, they improved PVI firing and reversed behavioural impairments [156]. In healthy human participants, a Kv3-modulator (AUT00206) attenuated ketamine effects on fMRI BOLD activation in the ACC, precuneus and thalamus [157]. A PET study found that Kv3-modulation had no overall impact on dopamine synthesis capacity in patients with SSD [158]. However, there was a significant positive correlation between dopamine synthesis capacity reduction and positive symptom reduction in the group that received the Kv3-modulator.

#### Glutamatergic mechanisms

Hypofunction of NMDAR on GABAergic neurons may lead to loss of PVI activity in the hippocampus and PFC. Medications that increase the activity of NMDAR without leading to excitotoxicity have therefore been investigated.

NMDAR co-agonists targeting the glycine binding-site (glycine, d-serine, d-cycloserine and d-alanine) showed promise in small early trials [159, 160], but failed to meet clinical endpoints in phase III trials [161]. A meta-analysis of adjunctive treatment in patients with persistent negative symptoms found no benefit for d-cycloserine, while the combination of glycine or d-serine with d-cycloserine showed a small improvement in negative symptoms [162]. A large multicentre trial showed no significant improvement in negative or cognitive symptoms with glycine or d-cycloserine [161]. In a small sample of CHR-P individuals, d-serine resulted in a significant reduction in negative symptoms [163], which requires confirmation in larger samples. Small EEG studies have found improvements in mismatch negativity frequency with acute administration of glycine [164] and 6 weeks of d-serine [165] in SSD, but pharmaco-neuroimaging evidence is limited for these compounds.

Selective glycine transporter inhibitors have also been evaluated. Biopertin resulted in a significant reduction in negative symptoms in a phase II trial [166], but later trials found no significant improvement in patients with prominent negative symptoms [167]. Iclepertin showed significant improvements in cognitive symptoms in a phase II study [168], but the relationship between the treatment response and EEG measures, including mismatch negativity and resting gamma power, were modest and inconsistent [169]. There are phase III trials ongoing [170]. Sarcosine was also promising in initial trials [171, 172], but in larger trials, there were no symptom improvements [173]. However, there were improvements in cognitive symptoms when sarcosine was combined with benzoate [173], which inhibits the enzyme that metabolises D-serine. In small studies, benzoate reduced negative and cognitive symptoms in chronic [174] and clozapine-resistant SSD [175]. A recent meta-analysis found that adjunctive benzoate improved positive symptoms but had no effect on negative symptoms or cognition [176]. There was a significantly higher occurrence of extrapyramidal symptoms with benzoate, which raised safety concerns [176]. Imaging studies with benzoate have been limited; a small study with individuals with mild cognitive impairment reported decreased fMRI regional homogeneity in the right orbitofrontal cortex after benzoate treatment [177].

Meta-analyses with memantine, a low-affinity non-selective NMDAR antagonist, have had mixed results (reviewed [178]), with the most recent reporting improved negative and cognitive symptoms in chronic SSD [179]. Due to strong voltage-dependency and rapid channel-blocking, memantine can block pathological NMDA activity while maintaining signalling related to cognitive functioning [178]. EEG studies found that a single dose of memantine normalised gamma oscillatory power and phase synchrony in SSD [180, 181]. An ongoing study in antipsychotic-naïve first-episode psychosis will investigate its effects on regional glutamatergic metabolites and structural brain changes [182]. Memantine has generally been reported to be well-tolerated in clinical practice, with low rates of central nervous system side effects [178]. This is thought to be due to its low affinity, fast off-rate and preferential inhibition of extra-synaptic receptors [178]. Further research is needed to establish whether there is a role for memantine in the treatment of SSD, possibly in combination with other medications acting on the NMDAR.

By depolarising neurons, allosteric modulators of AMPAR can remove the magnesium block of NMDAR, therefore increasing the availability for glutamatergic signalling. CX-516 showed some promise as an adjunctive treatment with clozapine for cognitive symptoms [183], but in a larger study, there were no significant improvements when added to clozapine, olanzapine or risperidone [184]. Similar compounds (piracetam and diazoxide) resulted in positive symptom improvements in small trials [185, 186]. Overall, there is limited evidence for these compounds and a lack of pharmaco-imaging studies.

Another approach to decrease the excitability of pyramidal neurons is reducing pre-synaptic glutamate release via positive allostatic modulation of metabotropic receptors. mGluR2/3 is highly expressed in limbic areas and regulates excessive glutamate release [187]. mGluR2/3 agonists reduced hippocampal activity and atrophy in pre-clinical studies [25], and reduced ketamine associated BOLD fMRI increases in healthy individuals [188, 189]. Pomaglumetad methionil reduced positive symptoms in initial studies but failed in phase II/III trials [190]. Re-analyses suggested that pomaglumetad may be effective in earlier psychosis stages [191]. Other mGlu2-specific compounds (e.g. AZD8529 and LY2979165) showed promise in phase I trials and pharmaco-fMRI studies [189, 192], but had negative results in other trials [193].

Riluzole prevents glutamate release by inhibiting voltage-gated calcium channels and increasing the uptake of extracellular glutamate by astrocytes [194]. It also increases the depolarising effect of GABA and interacts with dopamine and acetylcholine [194]. A small clinical trial in SSD found that adjunctive riluzole reduced negative symptoms [195]. In treatment-resistant SSD, a 2-day riluzole challenge led to a reduction in glutamate + glutamine concentration and increased ACC-anterior PFC connectivity [196]. Higher baseline glutamate + glutamine concentrations were associated with more severe negative symptoms. A more recent review with treatment-resistant participants also found increased frontal connectivity after riluzole treatment [197].

### Anticonvulsants

Several anticonvulsant medications have been investigated for their potential role in treating SSD. These medications have complex actions on multiple targets, but several act on voltage-gated sodium channels and reduce synaptic release of glutamate (e.g. lamotrigine, carbamazepine, topiramate). A meta-analysis of lamotrigine in SSD found small improvements in negative, cognitive and positive symptoms, but these are unlikely to be clinically significant, and the evidence was not robust [198]. Topiramate has a broad range of actions, including AMPA/kainite receptors, calcium/sodium channels and blocking GABA reuptake [199]. Several meta-analyses indicate topiramate improved all symptom domains, but these findings are considered preliminary, and there are safety concerns regarding possible psychiatric side effects [200, 201]; larger studies are thus needed to further investigate these findings. Imaging studies with topiramate have been limited to healthy participants and individuals with epilepsy or migraine; they have reported a pattern of reduced activation across the inferior and middle frontal gyri, and a reduced ability to deactivate task-negative networks [202, 203]. In healthy individuals, a study combining transcranial magnetic stimulation with sodium valproate/lamotrigine showed that lamotrigine increased connectivity between the dlPFC and the ACC when transcranial magnetic stimulation was applied to the PFC [204]. Meta-analytic evidence indicates that sodium valproate augmentation in SSD significantly improved total symptoms when open-labelled studies were included, but this was not significant when only RCTs were pooled [205].

Levetiracetam targets synaptic vesicle protein 2A and reduces pre-synaptic glutamate by regulating the expression/trafficking of synaptic vesicle sensor proteins and blocking voltage-gated calcium channels [206]. It also increases GABA receptor activation [206]. Levetiracetam was found to have significant effects on global symptoms, particularly negative symptoms, in a small RCT with individuals with SSD [207]. A recent neuroimaging study found increased resting-state fractional amplitude of low-frequency fluctuations (fALFF) in the hippocampus of patients with SSD, reflecting increased spontaneous neural activity in this region. Levetiracetam reduced hippocampal fALFF to a level that did not differ from controls [208]. Another study in medication naïve SSD reported that levetiracetam reduced hippocampal blood flow, but this non-crossover study was limited by very small sample sizes [209]. There are studies ongoing investigating the effect of levetiracetam on symptom domains and/or hippocampal hyperactivity in SSD (NCT04317807, NCT02647437 and NCT03034356) and CHR-P (NCT06224530). It will also be important to evaluate the safety profile of levetiracetam in SSD, given concerns regarding potential psychiatric side effects primarily from case reports and retrospective studies in epilepsy [210].

### Targeting oxidative stress and neuroinflammation

Given the evidence for increased oxidative stress and its impact on PVI function, there have been trials of antioxidants (e.g. *N*-acetyl-cysteine (NAC), vitamins C/E, allopurinol, omega-3-polyunsaturated fatty acids and ginkgo biloba [211]) and anti-inflammatory agents (e.g. minocycline, NSAIDs, monoclonal antibodies [212]) in SSD. While large meta-analyses suggested an overall improvement in psychopathology with some of these compounds, many studies were small, and the mechanisms likely extend beyond the direct anti-inflammatory/antioxidant effects [213].

NAC has been extensively investigated in patients with SSD. NAC has multiple protective mechanisms, including: increasing glutamate transport into glial cells, increasing extra-synaptic glutamate release via activation of mGlu2/3, and antioxidant effects as the precursor to glutathione [214]. Meta-analyses indicate superiority to placebo in all symptom domains, although the RCTs were small and heterogeneous [215, 216]. The effect size was larger for patients in the acute phase of illness and at longer treatment durations (>24 weeks). Despite this, another meta-analysis reported no significant difference in any symptom domain in both early (<24 weeks) and late (>24 weeks) timepoints [217]. Pharmaco-imaging studies in patients with SSD found that NAC modulated EEG synchronisation over the left parietal-temporal, right temporal and bilateral PFC regions [218] and reduced fMRI resting-state connectivity in medial frontal areas [219]. Changes in structural connectivity have also been demonstrated after 6 months of NAC, with an 11% increase in fornix white matter integrity [220]. NAC increased peripheral and MRS mPFC measures of glutathione in early psychosis [221] and in chronic SSD [222], therefore, peripheral redox markers could be used to stratify patients into subgroups that will benefit more from NAC supplementation [221].

Targeting the mitochondria more directly with mitoquinone mesylate (MitoQ) can rescue oxidative stress and mitochondrial damage in animal models [223]. Alterations in oxidative stress blood markers have been identified in early psychosis, and these marker differences correlated with reductions in auditory response gamma oscillations in EEG [223]. As a subgroup of SSD with high mitochondrial dysfunction and more severe symptoms can be identified using these markers [223], a clinical trial is ongoing to test if these mechanism-based biomarkers could be used to stratify patients most likely to respond to MitoQ (NCT06191965).

### Targeting the other interacting systems

Although we have focused on the role of excitation/inhibition balance in the corticolimbic system we acknowledge that there are complex interactions with other systems, including serotonin, acetylcholine and endocannabinoid systems. A comprehensive review of compounds targeting these systems is beyond the scope of this review, but we briefly outline results that are relevant to the corticolimbic system in Table 1.

**Table 1:** Summary of medications targeting interacting systems with relevance to the corticolimbic system in Schizophrenia Spectrum Disorders (SSD).

Medications (Mechanism)	Corticolimbic pharmaco-neuroimaging	Impact on symptoms
***Endocannabinoid***		
Cannabidiol (multiple targets)	• *Healthy Participants*- Decreased corticolimbic activity in healthy individuals [237, 238]. • *SSD*- Decreased hippocampal and parahippocampal activity. Normalised hippocampal-striatal functional connectivity during verbal memory recall [239].	• *CHR-P-* not effective when given for short durations. Longer durations may be needed [240, 241]. • *SSD*- improvements in PANSS positive scale and clinician’s overall impression (CGI-I) with 6 weeks adjunctive treatment [242].
***Acetylcholine***		
Xanomeline (muscarinic M1 and M4 agonist)	• *Rodents -* Dose-dependently attenuated ketamine-induced brain activation [243]. Increased neocortical-striatal connectivity and attenuated corticolimbic response and frontal-hippocampal hyperconnectivity induced by PCP [244]. • *SSD-* Lower [3H]pirenzepine binding in the cortex, hippocampus and striatum [245].	• *SSD-* pilot study (*N* = 20) improved BPRS and total PANSS scores [246]. High rates of adverse effects.
KarXT (xanomeline-trospium)	N/a	• *SSD-* Phase II studies- improved PANSS total scores [247]. Phase III studies- improved PANSS total score at 5 weeks [248] Ongoing studies (NCT05145413, NCT04738123, and NCT04659174).
Emraclidine CVL-231 (M4 positive allostatic modulator)	N/a	• *SSD-* Phase 1B study- decreased total and negative PANSS [249]. Phase II ongoing (NCT05227690).
EVP-6124, AVL-3288 (α7 nicotinic receptor partial agonists/positive allostatic modulator)	N/a	• *SSD-* Phase 1b study- non-significant worsening in RBANS scores [250]. Phase III study- no significant benefit on cognitive symptoms [251]. Metanalysis- not effective for cognitive symptoms and small effect for negative symptoms [252].
***Serotonin***		
Pimavanserin/ ACP-103 (inverse agonist/ antagonist 5-HT_2A_ receptors)	• *Rodents-* Increased dopamine release in mPFC [253].	• *SSD-* Adjunctive use with low dose risperidone improved PANSS total score [254]. Phase II study - improved negative symptoms [255]. Phase III study - no significant difference in PANSS total scores [256].
Roluperidone/ MIN‐101 (5‐HT2A and sigma‐2 antagonist)	N/a	• *SSD-* Phase II study- no significant reduction in PANSS total or cognitive symptoms [257]. Phase III study- improvements in negative symptoms (NSFS) [258].

Evidence from schizophrenia spectrum disorders (SSD), clinical high risk for psychosis (CHR-P), healthy participants and rodents. Scales- positive and negative syndrome scale (PANSS), The clinical global impressions scale- improvement (CGI-I), brief psychiatric rating scale (BPRS), repeatable battery for the assessment of neuropsychological status update (RBANS) and negative symptom factor scale (NSFS).

### Non-pharmacological approaches

Non-invasive brain stimulation has potential benefits in improving corticolimbic system function. In one of the few studies investigating neural changes in SSD with transcranial direct current stimulation, left dlPFC stimulation led to significant increases in gamma synchronisation [224]. Reviews suggest that transcranial magnetic stimulation and transcranial direct current stimulation may have promise in managing negative and cognitive symptoms in psychosis, but the evidence is mixed, and the studies are heterogeneous [225, 226].

Cognitive training involves performing cognitive tasks which are intended to improve function by activating relevant neuronal circuits and altering plasticity. A meta-analysis of neuroimaging studies with cognitive training showed that increased left PFC activation was most frequently observed, but there were effects on many regions, possibly due to heterogeneous training programmes [227]. Gamma oscillatory power has been shown to predict cognitive improvement after a course of cognitive remediation [228]. Combining cognitive training with either pharmacological interventions [229] or non-invasive brain stimulation may enhance the pro-cognitive effects [230].

## Challenges and opportunities

Despite progress in understanding the role of corticolimbic circuit disruption in SSD and its potential therapeutic manipulation, promising results from pre-clinical and early clinical studies have largely not translated in later-phase clinical trials. There are multiple factors contributing to translation barriers, including intervention timing, target population heterogeneity, lack of biomarkers and lack of medication selectivity.

Many of the studies discussed were conducted with individuals with established SSD who have been treated with antipsychotics for many years, sometimes with a brief washout period. Antipsychotics exert long-term effects on neurotransmitter systems and brain circuits, which may be sufficient in making novel agents non-effective [231]. For instance, compensatory brain changes due to D2 antagonists can lead to increased sensitivity to dopamine receptor stimulation, known as dopamine receptor supersensitivity [232]. Pre-clinical studies have shown that haloperidol pretreatment-induced D2 supersensitivity which masked the efficacy of a GABA_A_-α5 compound [231]. There is evidence that active psychosis is also harmful to prognosis, although evidence of global neurobiological changes with untreated psychosis is more mixed [233]. Addressing these challenges could involve testing novel drugs in patients who have been on medications which are less likely to produce supersensitivity, such as partial dopamine agonists, and early interventions or preventative methods in at-risk/early psychosis populations.

Adolescence/early adulthood is a critical period due to multiple neurobiological and environmental factors. Heightened plasticity and perineuronal net development during this time makes these structures susceptible to oxidative stress, which may lead to PVI dysfunction [234]. Animal models with peripubertal administration of pharmacological treatments, including antioxidants, glutamate or GABA modulating agents, have been shown to prevent perineuronal net damage, PVI loss and increased dopaminergic activity [25, 33]. This suggests that interventions targeting excitation/inhibition imbalances and oxidative stress may have preventive potential in SSD, although the long-term broad administration of some of these medications would not be feasible in at-risk human populations due to poor tolerability. Longitudinal research tracking the pathological changes in corticolimbic circuits could help to identify the optimal timing for different interventions.

Another major obstacle to translation is the continued reliance on diagnostic categories, disregarding the considerable pathophysiological and phenomenological heterogeneity in SSD. Meta-analyses typically examine the entire patient population, potentially overlooking treatment response in specific subgroups. Patient stratification based on relevant biomarkers would enhance group homogeneity, but this relies on identifying appropriate predictive markers of treatment response. Neuroimaging methodologies provide a promising route for identifying biomarkers, but scalability issues may hinder clinical application [235]. Some progress has been made in identifying blood makers, such as for oxidative stress and mitochondrial function [221, 223]. Promising new evidence also suggests that increased peripheral blood levels of endothelial glycocalyx, which indicates blood–brain barrier dysfunction, can differentiate first-episode psychosis from healthy controls and is associated with increased symptom severity [236]. Despite these advances, targeted treatment approaches remain in the early stages of development in SSD research.

Many of the novel pharmacological approaches outlined above interact with several neurotransmitter and receptor subtypes, both within and beyond the corticolimbic system. This lack of selectivity makes it challenging to distinguish which mechanism is beneficial and why. The development of compounds with greater regional and subunit specificity would help untangle this complexity. A better understanding of the neurobiological mechanisms that underlie the considerable heterogeneity in psychosis will also help identify the most effective interventions for the individual. Neuroimaging combined with pharmacological and non-pharmacological interventions can enhance our understanding of how molecular targets impact wider neurocircuits. It is also important to integrate different research modalities to test hypotheses across computational, cellular, animal and human scales.

## Conclusions

The progress that has been made in understanding the role of the corticolimbic system in SSD holds great promise for future successful translation of treatments. Further research to better understand why many of the novel compounds have failed and the role of illness stage and prior treatment exposure will be essential to progressing drug discovery. Combining multiple modalities to examine the same hypotheses can help to increase our understanding of the neuropathological processes within the corticolimbic system. For instance, utilising neuroimaging across species can explore homology between circuits and validate theories via back translation. The combination of neuroimaging measures with experimental interventions can substantially improve our understanding of how molecular modulation influences wider corticolimbic function and behaviour/symptomatology. Large-scale consortia afford the power needed to use multivariate data-driven methods and normative modelling to make inferences at the individual level. Increasing our knowledge of biomarkers and their use to stratify patient samples will also be integral to moving towards a precision medicine approach in SSD.
